# Selections and Abstracts

**Published:** 1899-06

**Authors:** 


### *SELECTIONS AND ABSTRACTS.*

### THE TREATMENT OF HEADACHE.

Dr. Joseph Collins writes an article on this practical subject in the *Medical News* of February 18, 1899. He says that those headaches accompanying the infectious diseases do not call for any particular treatment aside from the measures taken to combat the infectious processes, while the treatment of headache due to the ingestion of vegetable or mineral poisons resolves itself into the very simple matter of preventing the further inhibition of the poison, be it tea, alcohol, tobacco, or poisonous substances administered therapeutically or encountered in occupations, and the elimination of any of the poisons remaining in the system from the body. After that the headache disappears on the restoration of general, including neutral, nutrition. To bring about this restitution of nutrition general tonic treatment is required. And meanwhile the headache may be relieved temporarily by the administration of some of the the prescriptions possessing analgesic properties. A formula that Dr. Collins often uses as a general tonic and stimulant in headaches following the infectious and exogenous intoxications is as follows :

Opii pulv.............................................gr. ss.
Zinci phosphid........................................gr. ss.
M. Ft. pil. No. 20. Sig.: One pill three times a day.

Dr. Collins says that the tonic effect which one gets from this small quantity of opium is nothing less than remarkable. All the infections and intoxications, without exception, produce a more or less prefound condition of general anemia, and this anemia must be reckoned with in estimating the nature and determining the treatment of the headache. Early in the treatment of such headaches some such searching tonic as the following should be administered :

℞ Ferri et ammonii citratis..............................grs. xl.
Liq. potassii arsenitis.................................*m* xl.
Syr. zingiberis.........................................f ℥ ss.
Inf. calumbæ............................................ad f ℥ iv.
M. Sig.: Two tablespoonfuls after meals.

He is especially apt to give this mixture to children who complain of headaches following the infectious diseases, while for adults the mistura ferri ammonii acetatis is substituted for the citrate salt.

In the treatment of headaches resulting from the absorption into the system of some endogenous poison, such as that of diabetes, uremia, and the autointoxicants and infections, the general measures to be adopted do not differ materially from those already spoken of. The headache is combated when the formation of the poison and its absorption into the system is interfered with. In this way diabetic headaches are treated by diet and by the utilization of remedies against the anemia and oligocythemia, while uremic headache is combated by measures that prevent the formation of urea, and by those that facilitate its excretion. In uremic headaches accompanying chronic interstitial nephritis of slow progression, Collins uses the following prescription as a diluent and diuretic with good effect:

℞ Potassii citratis........................................f ʒ ij.

Tinct. hyoscyami........................................f ʒ ij.

Spt. etheris nitrosi........................................f ʒ ij.

Inf. scopariæ........................................f ℥ vj.

M. Sig.: Tablespoonful in water three times a day.

If it is necessary to increase vascular tension, infusion of digitalis may be added to this mixture.

Headaches arising from such intoxication as that of ammoniemia require only local treatment of the cystitis and the institution of measures to combat the anemia.

Headaches arising from autointoxication, the original source of the disease being stomachic and intestinal catarrh, functional perversion of the glands supplying the digestive juices, or through the activity of non-pathogenic bacteria, taken in from outside, form an important class, and one that is happily rather amenable to treatment. It must suffice in this connection to say that after the general measures for the regulation of the alimentary tract and its associated functional dependencies, such as the overcoming of constipation, the adoption of suitable diet in catarrhal conditions, the stimulation of the liver to the production of a suitable kind and amount of bile, the exhibition of substances that contribute to the restoration of the pancreas and spleen, the treatment consists in the administration of substances that correct the apparent troubles of digestion and of the substances that quell the headache. A favorite prescription of Dr. Collins's for headache associated with flatulency and pyrosis is the following:

℞ Sodii bicarb.,

Bismuthi subgallate,

Pulv. acaciæ, each............................ ʒ j.

Liq. ammonii anisi..............................f ʒ ij.

Aqua dest... ..................................ad f ℥ viij.

Sig.: Two teaspoonfuls before meals, repeated in three hours if necessary.

In headaches associated with atonic dyspepsia but without any considerable flatulency, Collins makes use of the following pills, and especially in the headaches occurring in women:

℞ Ferri sulphatis,
Quininæ sulphatis, ää.................. ............grs. xv.
Sodii arsenitis....................................gr. ss.
Pulv. rhei.,
Pulv. zingiberis, ää............... ......... .....grs. x.
M. Ft. pil. No. 12. Sig.: One pill three times a day after meals.

The treatment of headaches due to disease of the circulatory system requires considerable discussion, not so much because of their frequency as because of the fact that if they are properly interpreted they yield readily to treatment. The headaches that accompany organic diseases of the heart, whether they be associated with excess or deficiency of propulsive power, naturally require treatment directed to that organ, as does any pulmonary condition which interferes with the return cerebral circulation. Headaches occurring with functional disturbances of the heart are oftentimes very amenable to therapeutic measures, not drugs. For instance, a heart that is working violently as the result of great physical effort or excitation of mind or body may be so quieted by the application of a simple cold-water compress to the cardiac region that the accompanying frontal throbbing headache disappears promptly, and the efficaciousness of stimulating foot-baths and hot sitz baths in combating a headache of increased vascular tension within the skull is very well known. It is rarely necessary to administer the more powerful cardiovascular depressants in cases of this kind, the required equalizing of the circulation being obtained by hydric procedures and the administration of a few doses of the bromides. When headache is an accompaniment of a sluggish circulation, there being no deficiency in the amount of the blood and no alteration of its constitution, the diffusible stimulants, caffeine and strychnine, may be relied upon to bring about its prompt relief. Cannabis indica is a drug that he frequently uses with good effect in this form of headache. The method of prescribing it is in the following pill:

℞ Ext. cannabis indicæ.......................... gr. ⅓ to ss.

Ext. gentianæ.. ................................q. s.

M. Ft. pil.

Headaches that are dependent upon a general anemia are oftentimes extremely resistant to treatment, and although temporary improvement usually follows tonic and stimulating treatment, the anemia must be fought unswervingly for a long time to effect a complete cure and to stay the recurrence of the headache. These headaches are usually accompanied by a very slight sluggish condition of the digestive tract, to combat which he has used with very good results the following combination of tonics and laxatives in the shape of a dinner pill:

℞ Quininæ sulph.,
Ext. aloes aq.................................ää grs. xij.
Pulv. capsici.,
Pulv. ipecac..................................ää grs. vj.
Glycerini.....................................q. s.
M. Ft. pil. No. 12. Sig.: One pill at midday.

Or, if associated with considerable vital depression, he uses the following pill instead, giving at the same time some absorbable form of iron:

℞ Ext. nucis vomicæ...... ............................gr. ss.
Pil, rhei comp.................................. gr. iij.
Pulv. capsici.........................................gr. ¼
M. Ft. pil. Sig.: One pill at midday.

Naturally it is very often necessary to give at the same time, for its immediate effect, some analgesic or a combination of these with a stimulant, such as caffeine, and such a prescription as one given above, containing caffeine, phenacetine and salol, usually meets the requirements.—*Therapeutic Gazette.*

---

### MANAGEMENT OF PATIENTS BEFORE AND AFTER LAPAROTOMY.

In the course of an interesting and practical paper with the above title, read before the New York Medico-Surgical Society, by Dr. Frederick Holme Wiggin, Visiting Gynecologist to the City Hospital; Surgeon to St. Elizabeth's Hospital, it is advised:

"If the operation is to be performed at an early morning hour (*i. e.*, 8 A.M.), the patient should be given a peptonized milk punch at 11 o'clock the previous evening, and if he awakens at 5 or 6 A.M. one ounce of Liquid Peptonoids may be given, but nothing more. It has been the writer's experience that a small amount (one ounce) of stimulating and concentrated food, administered about two hours prior to the taking of the anesthetic, diminishes the liability to heart failure, and also lessens the nausea and vomiting which frequently follow the return to consciousness. If an afternoon hour has been decided upon, another peptonized milk punch may be given the patient when he awakes, and one ounce of Liquid Peptonoids at 11 A.M."

In considering the after treatment, the distinguished author says:

"With the passing of the first twelve or eighteen hours, if the patient is not suffering from nausea or vomiting, and the pulse rate is much the same as before the operation, a drachm of Liquid Peptonoids or of some other similar preparation may be given, and repeated, if well borne, every twenty minutes until four doses have been taken, when after an interval of two hours a small quantity of equal parts of milk and lime water or of peptonized milk may be given from time to time, until four ounces have been taken. After this there should be an interval of two hours and then half an ounce of Liquid Peptonoids may be administered."

---

**NOTICE.—This JOURNAL and THE INTERNATIONAL JOURNAL OF SURGERY one year for $1.00 cash, to new subscribers. No out-of-town checks accepted unless 10c. is added for cost of cashing.**

### THE THERAPY OF CARCINOMA OF THE RECTUM.

Dr. Hochenegg has operated in 129 cases of rectal carcinoma since 1890; of these thirty-four were colostomies, eighty-nine sacral extirpations, and six perineal amputations (*Wiener klinische Wochenschrift*, 1897, No. 32). Of the eighty-nine sacrally-operated cases eight were fatal, though three of the deaths had no connection with the operation. This is certainly a magnificent record. The writer refuses operation in cases of marked general debility, and when there are symptoms of internal metastases; in cases in which the tumor is immovably fixed in the pelvis; and when the glandular infection cannot be limited by the examining finger. Adhesions to the prostate, bladder, vagina, or uterus form no contraindication to a radical operation, since these organs can, if necessary, be extirpated. In doubtful cases a colostomy is at first performed; two weeks later, when the tumor becomes less fixed, the extirpation is done. Symptoms of acute intestinal obstruction also form a contraindication to radical operation. Radical extirpation may be performed by either of two methods, different in principle: perineal and sacral. Hochenegg applies the perineal method in those cases also in which the carcinoma has not involved the anal portion very high up and in which the rectal mucous membrane is soft and easily movable over the tumor. In all other cases he prefers the sacral operation. His procedure in the sacral operation is as follows: With the patient on the left side, a convex incision is made—with the convexity towards the right—from the left sacroiliac synchondrosis to the right lateral border of the coccyx. After the skin has been raised the coccyx is extirpated and the left wing of the sacrum is chiseled off; in extensive carcinomatous disease the sacrum is severed transversely. Further procedure depends upon the location and extent of the tumor. If the anal portion be also involved an incision must be made around this portion; the rectum is then isolated and amputated to a point above the site of the neoplasm; the lumen of the remaining rectum is drawn down and sewed in the place of the removed anus, or is brought under the skin at the site of the removed sacrum (anus præternaturalis sacralis); the writer prefers the latter method. If

5

the anal portion be not diseased and the sphincter intact the severed rectum and the healthy anus are sewed together; this is the "ideal" operation. To prevent separation of the line of suture, because of the insufficiency of the circular stitch, Hochenegg proceeds as follows: The tumor is isolated and cut off above, and is then isolated below, but not yet amputated. The healthy rectum above is then isolated and the amount of its mobility is tested. If this portion of the rectum can be easily brought to the anal opening the tumor is cut off transversely at a point one centimeter above the sphincter, the anal portion everted and freed of its mucous membrane with forceps and scissors; through the tubular wound surface thus produced the healthy rectum above is drawn and is sutured *in situ*. There are two rows of sutures: one introduced from the outside, in front of the anus; the other introduced through the sacral wound. Finally, the ring of the sphincter is connected with the rectum by several buried sutures. If the healthy rectum above is not sufficiently movable to be brought so far down, less of the anal portion must be sacrificed. If the rectum cannot even for a short time be brought through the anal opening, nothing is left but to introduce the sutures through the sacral wound. After the introduction of the sutures the writer leaves enough of the wound open to secure drainage. The bowels are moved after the fifth day of operation. At the slightest sign of phlegmonous inflammation the whole wound is immediately reopened and drained.—*Med. Record.*

---

### THE VALUE OF REST IN THE TREATMENT OF CARDIAC DISEASE.

If there is one fault in therapeutics to-day above all others it is the habit on the part of some physicians of employing drugs when other remedial measures will produce equally good results; and it is so much easier to write a prescription than to take the trouble to go into a minute detailed description of a line of treatment which is to be carried out, that in many instances the patient receives a medicine and does not receive salutary advice. Perhaps no better instance of the needless use of drugs, when other remedial measures will do equally well, can be deduced than the constant habit of some physicians of prescribing digitalis or other cardiac stimulants to patients who are suffering from various forms of cardiac disease, and too often a few minims of some powerful drug is given three times a day when hygienic measures would benefit the patient very much more.

One of these hygienic measures more frequently employed in England than in this country is rest in the treatment of valvular disease of the heart. While it is, of course, true that it is impossible to give to the heart-muscle absolute rest, it is nevertheless possible to protect it from a large amount of unnecessary wear and tear, and we have come to regard absolute rest in bed with massage and gentle Swedish movements as being of far greater benefit to most patients who are suffering from valvular disease with associated failure of the circulation than is digitalis or any similar drug.

Those who have ignored this important factor, rest, in the treatment of cardiac disease will, we are sure, be greatly surprised at the relief of the symptoms, both acute and chronic, which have brought the patient into professional care, even though no drug be given. In some instances it is wise to begin the treatment, after the patient has had twenty-four hours' rest in bed, by the administration of some fairly active cathartic which will unload the liver, which is very apt to be engorged with blood in cases of failure of circulation, and also to administer tonic drugs, such as minute doses of quinine and arsenic, for their beneficial influence upon the blood itself and upon nutrition in general. The patient ought to be sponged freely with alcohol and water, with active friction, preferably at night just before going to sleep, as this sponging will keep the skin clean, improve the peripheral circulation, and by allaying peripheral nervous disturbances cause the patient to sleep more comfortably.

Where the action of the heart remains excited and transmits a heaving impulse to the chest-wall, advantage may be gained by applying to the patient's precordium an ice-bag partly filled with small pieces of ice, which, because of its being partly filled, fits snugly to the chest-wall and does not readily fall off when the patient changes his position slightly.

Sometimes it is necessary, instead of administering small doses of digitalis, to give these patients small doses of aconite to put aside excessive cardiac action.—*Therapeutic Gazette.*

---

### THE REPORT OF THE WAR INVESTIGATION COMMITTEE.

The Commission arrived at the following conclusion in regard to the Medical Department during the war with Spain :

1. That at the outbreak of the war the Medical Department was in men and material altogether unprepared to meet the necessities of the army called out.

2. That as a result of the action through a generation of contracted and contracting methods of administration, it was impossible for the department to operate largely, freely and without undue regard to cost.

3. That in the absence of a special corps of inspectors, and the apparent infrequency of inspections by chief surgeons and of official reports of the state of things in camps and hospitals, there was not such investigation of the sanitary conditions of the army as is the first duty imposed upon the department by the regulations.

4. That the nursing force during the months of May, June and July was neither ample nor efficient, reasons for which may be found in the lack of a proper volunteer hospital corps, due to the failure of congress to authorize its establishment, and to the nonrecognition, in the beginning, of the value of women nurses and the extent to which their services could be secured.

5. That the demand made upon the resources of the department in the care of sick and wounded was very much greater than had been anticipated, and consequently, in like proportion, these demands were imperfectly met.

6. That powerless as the department was to have supplies transferred from point to point, except through the intermediation of the Quartermaster's Department, it was seriously crippled in its efforts to fulfil the regulation duty of "furnishing all medical and hospital supplies."

7. That the shortcomings in administration and operation may justly be attributed, in large measure, to the hurry and confusion incident to the assembling of an army of untrained officers and men, ten times larger than before, for which no preparations in advance had been or could be made because of existing rules and regulations.

8. That notwithstanding all the manifest errors, of omission rather than of commission, a vast deal of good work was done by medical officers, high and low, regular and volunteer, and there were usually few deaths among the wounded and the sick.

The Commission makes definite recommendations for the future conduct of the Medical Department, urging the necessity of a larger force of commissioned medical officers; authority to establish in time of war a proper volunteer hospital corps; the establishment of a reserve corps of selected trained women nurses; extra supplies of all sorts to be held constantly on hand; improvement in transportation; less red tape; the authorization of surgeons to purchase such articles of diet as may be necessary for the proper treatment of soldiers too sick to use the army ration.

On the whole, we must regard the report as a fair statement of the conditions as derived from the evidence at the disposal of the Committee.—*North Carolina Medical Journal.*

---

### PREVENTION OF HAY FEVER.

In the January 21, 1899, number of *The Journal of the American Medical Association*, Dr. Alexander Rixia of New York, contributed a very interesting article on "Prevention of Hay Fever." After a highly interesting historical review, and a brief survey of the results achieved in the past few years, the writer resumes the results of his own investigations.

His ingenious researches for a number of years, regarding the etiology of hay fever, lead him to admit that the pollen of the Roman wormwood, ragweed (*ambrosia artemisœfolia*) is the primitive and active cause of this peculiar disease. By inhaling these pollen he produced the symptoms of genuine hay fever. He writes as follows:

From the time I found the pollen to be the exciting cause of the disease, I concluded in a logical way upon the proper treatment. I conceived the idea of rendering the receptacle aseptic by preparing the soil for the reception of the pollen. Naturally, they will find no proper soil for a possible generation, propagation or development, destroying their existence in embryo, so to speak, and with it the real cause of hay fever. For this purpose I decided on the following treatment:

About two weeks before the onset of the disease I commence to irrigate or sterilize the nasal cavity and the post-nasal spaces with a harmless antiseptic solution, using the douche and atomizer. After giving a great number of antiseptics a fair trial, I decided on Hydrozone as the most innocuous and most powerful germicide. Hydrozone is a 30-volume aqueous solution of peroxide of hydrogen. At the beginning I use it for irrigation diluted in the proportion of one ounce of Hydrozone to twelve ounces of sterilized water. Nearing the period of the expected onset of the disease I increase the dose to two or three ounces of Hydrozone to twelve ounces of the sterilized water, according to the severity of the disease, using the douche, either tepid or cold, four times a day—morning, noon, evenings and at bedtime—while during the intervals I use the atomizer, with a solution of Hydrozone and pure glycerin, or sterilized water, one to three, thus keeping the nares perfectly aseptic during the entire period, and preventing the outbreak of the disease in consequence thereof.

In most obstinate cases, when there is still some irritation in the nasal cavity, I give as an adjuvant the following prescription:

℞ Acid boracic.................................... gr. xx.
Menthol..........................................gr. iv.
Glyco-thymoline................................℥ij.
Sol. eucain B. 4 per cent........................q. s. ad ℥ ij.
Sig.: Use in atomizer.

As a rule this treatment was sufficient to avert the disease and keep the patient in perfect comfort.

### A MEDICAL CABINET OFFICER.

Dr. J. D. Emmet, the very able editor of that most excellent publication, *The American Gynecological and Obstetrical Journal*, has come out in a strong editorial advocating a medical representative in the President's Cabinet. He asks the medical press of America to support his position and this journal gladly accedes to his request.

Probably the question has many times come in a vague way to the minds of most medical men. Those who have not pondered the matter, and those who are not convinced of its necessity and its justice, will require but a few words to bring conviction. There is a cabinet officer for almost every public necessity and utility excepting that of medicine and the public health. There is at the same time nothing so important as the health of the people. Our foreign relations, our finances, our interior affairs, the post-office, the law, the army, the navy, agriculture, all are important and must be regulated and administered; and yet the public health, the influence which affects every family, every individual, more important than any or all other agencies combined, that is the one neglected and ignored.

In this day of keen competition among manufacturers, and the eager search for wealth, the adulteration of food and drugs is widely and unscrupulously practiced. The shameful treatment of our soldiers within the last year, and the deplorable results which brought many more deaths from food than from fighting, could have been avoided had a competent and conscientious medical cabinet officer directed the choosing and the distribution of the rations.

We have no national health department. The control of our ports, the health of the army and navy, the prevention and suppression of epidemics, are under the direction of either a non-medical man or of a subordinate. In all questions of large importance the President has an able counselor of his own choosing, with the one exception of the public health.

Medical men throughout the country should take this matter in hand and vigorously press it to a successful conclusion. This journal invites correspondence on the question, and will gladly publish whatever the profession may have to say.—*Pacific Medical Journal.*

### ALCOHOL AS AN ANTIDOTE FOR CARBOLIC ACID.

Occasional reports of carbolic acid poisoning, which have appeared in recent issues of the current medical press, remind us of the success achieved by Phelps in antidoting carbolic acid by the use of alcohol. He states that the hands may be washed with impunity in ninety-five per cent. carbolic acid and that no escharotic effect will result if they be immediately dipped in alcohol. He has employed injections of pure carbolic acid in suppurating cavities and has then washed them out with alcohol. The procedure has not been accompanied by carbolic acid intoxication. The method has been found to be very efficient in immediately sterilizing suppurating cavities, and many cases have been followed by a rapid absorption of the walls of the abscess and an obliteration of its cavity. The importance of the discovery in relation to accidents with carbolic acid cannot be overestimated. The frequency of accidental poisoning with this drug has greatly increased of late years, and the occasional accidental spilling of the contents of a bottle of strong carbolic acid over some portion of the body is by no means infrequent. The application of alcohol to these cases is said to furnish a perfect antidote. Carbolic acid, when swallowed, if followed at once by alcohol, is said to be immediately antidoted.

---

### A MENSTRUATING MAN.

Dr. Rushton Parker (*British Medical Journal*, April 1) records the case of a man, twenty-four years of age, who married a woman, but was unable to effect sexual intercourse with her. She noticed that he had a monthly sanguineous discharge lasting about three days and staining the linen. After eight months they consulted Dr. Parker. He describes the man as follows:

"I found nothing unusual in his general appearance, except a shy, cowed look; the penis, urinary meatus, and scrotum seemed normal, but the testes felt decidedly small and soft; he stated that he had never either abused himself or had any sexual feeling whatever. Being satisfied that he was at least not a complete man, and doubtless cowed by his wife's dissatisfaction, he readily consented to a separation and allowed her half his income."—*New York Medical Journal.*

